# Too Much Cleavage of Cyclin E Promotes Breast Tumorigenesis

**DOI:** 10.1371/journal.pgen.1002623

**Published:** 2012-03-29

**Authors:** Keith R. Loeb, Xueyan Chen

**Affiliations:** 1 Department of Laboratory Medicine, University of Washington School of Medicine, Seattle, Washington, United States of America; 2 Department of Pathology, University of Washington School of Medicine, Seattle, Washington, United States of America; 3 Division of Clinical Research, Fred Hutchinson Cancer Research Center, Seattle, Washington, United States of America; University of Washington, United States of America

Cyclin E, together with cyclin-dependent kinase 2 (CDK2), functions as a gatekeeper to promote G1/S transitions and the initiation of DNA replication. In normal cells, cyclin E–associated kinase activity is exquisitely regulated, with activity being limited to a brief time interval between late G1 and early S phase. Human cancers frequently exhibit deregulated cyclin E–associated kinase activity resulting from overexpression of cyclin E and loss of cyclin-dependent kinase inhibition (via p53 mutations) promoting genetic instability and cell proliferation [1]. Increased levels of cyclin E correlate with tumorigenesis and are a poor prognostic indicator independent of proliferation rate, suggesting that cyclin E's role in tumorigenesis is not limited to promoting increased cell proliferation [2], [3]. By eliminating regulatory constraints using p53 null cells, we and others have shown that overexpression or endogenous expression of stabilizing mutant forms of cyclin E can lead to hyperproliferation, genetic instability, and malignancy in cell culture and murine models [4], [5]. Normal cells suppress the effects of excess/stabilized cyclin E via the G1/S checkpoint involving the p53/p21 pathway.

**Figure 1:**
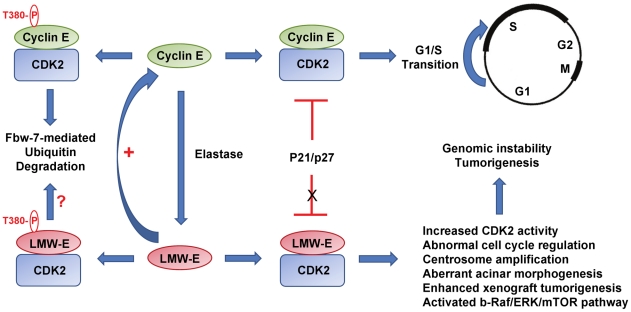
Low molecular weight cyclin E promotes tumorigenesis. In normal cells, cyclin E/CDK2 is tightly regulated and triggers the onset of S phase. In tumors, cyclin E undergoes proteolytic processing generating low molecular weight species that exhibit increased kinase activity and resistance to inhibition by cyclin kinase inhibitors p21/p27. The expression of LMW-E promotes aberrant acinar morphogenesis, centrosome amplification, and tumors associated with activation of the bRaf/ERK/mTOR pathway. LMW-E, low molecular weight cyclin E.

Five isoforms of cyclin E, ranging in size from 33 to 44 kDa, have been identified in tumors over-expressing cyclin E. These low molecular weight forms of cyclin E (LMW-E) are generated through post-translational cleavage of full-length cyclin E by the elastase family of serine proteases in tumor cells [6], [7]. In comparison to full-length cyclin E (50 kDa), LMW-E forms are uniquely expressed in tumor cells, exhibit enhanced CDK2-associated kinase activity, have increased affinity for CDK2 [7]–[9], and exhibit decreased inhibition by CDK2 inhibitors, p21 and p27 (Figure 1) [10], [11]. Ectopic expression of LMW-E isoforms promotes cell proliferation, genetic instability, centrosome amplification, and malignancy [12], [13]. In addition, clinical studies have shown that high LMW-E is strongly associated with poor survival in breast cancer [2], colorectal cancer [14], [15], ovarian cancer [16], and melanomas [17]. Given its unique properties and distinct function in human cancers, targeting LMW-E could have important therapeutic implications.

The study by Duong et al. in this issue of *PLoS Genetics* [18] convincingly uncovers the tumorigenic potential of LMW-E. The authors used three different model systems—3D acinar cultures, xenograft transplantation, and transgenic mice—to show that overexpression of LMW-E is sufficient to induce aberrant acinar morphogenesis in culture and mammary tumors in mice (Figure 1). When grown on Matrigel, immortalized human mammary epithelial cells (hMECs) expressing LMW-E exhibit large misshapen multiacinar structures resulting from defective growth arrest and apoptosis that mimic morphologic features of breast carcinomas. Further, ectopic expression of LMW-E in immortalized hMECs promotes tumorigenesis in xenografts and transgenic mice to a much greater extent than full-length cyclin E. Consistent with the reports by Akli et al. and Nanos-Webb et al., tumorigenesis associated with LMW-E is dependent on CDK2 [19], [20]. Furthermore, in vivo passaging of tumor cells increases the expression of LMW-E, suggesting that LMW-E provides a selective growth advantage to the tumor. Duong et al. also took advantage of a proteomic analysis termed reverse-phase protein array assay (RPPA) to examine protein expression patterns in cultured tumor cells and in breast tumors expressing high LMW-E levels. Their analyses revealed that multiple components of the b-RAF-ERK1/2-mTOR pathway are elevated in these cells. Activation of the b-RAF-ERK1/2-mTOR pathway normally promotes cell division and cell survival. Consistent with this, the authors observed that endogenous cyclin E levels are also increased in cells expressing high LMW-E, indicative of the existence of a positive feedback loop that promotes cell proliferation. Both high LMW-E levels and up-regulation of the b-RAF-ERK1/2-mTOR signaling pathway are associated with poor survival, suggesting functional correlation of these events in aggressive tumors. Importantly, the authors demonstrated that combination therapy targeting LMW-E/CDK2 and the b-RAF-ERK1/2-mTOR pathway has a synergistic effect in abrogating the tumorigenic effect of LMW-E. Thus, the identification of these downstream regulators may provide novel biomarkers and/or potential therapeutic targets for LMW-E–expressing tumors.

The report that LMW-E potentiates tumorigenesis in three independent model systems associated with activation of the b-RAF-ERK1/2-mTOR pathway is intriguing. However, there are many important questions about the role of LMW-E in tumorigenesis that need to be addressed. 1) What is the functional relationship between LMW-E and full-length cyclin E? In each tumor model reported by Duong et al., the effect was examined by over-expressing LMW-E in a background of endogenous full-length cyclin E. Further, the authors show that ectopic expression of LMW-E in transplanted xenografts triggers tumor evolution and results in increased levels of endogenous cyclin E. Thus, the contribution of endogenous full-length cyclin E in tumorigenesis cannot be excluded. In addition, Spruck et al. reported that the level of LMW-E correlates with full-length cyclin E, suggesting that LMW-E reflects the total cyclin E protein in primary breast tumors, cell lines, and even normal breast tissue [21]. To examine the effect of LMW-E in the absence of over-expression, and in the absence of full-length cyclin E, it will be important to use a knock-in model in which expression of LMW-E is driven from the endogenous cyclin E promoter. 2) What is the relationship between LMW-E and the b-Raf-ERK1/2-mTOR signaling pathway? The authors demonstrated that the b-Raf-ERK1/2-mTOR signaling pathway is activated in tumors expressing high levels of LMW-E. The b-Raf-ERK1/2-mTOR pathway may be a downstream signaling pathway deregulated by LMW-E, or it could be a parallel survival pathway selected in LMW-E–expressing tumors. In particular, the fact that only combinational therapy targeting both cyclin E–associated kinase activity and the b-Raf-ERK1/2-mTOR pathway generates an anti-tumor effect argues against a direct cause–effect relationship and is suggestive of a parallel pathway. 3) Is LMW-E expression required for tumor growth and does down-regulation of LMW-E alter tumor growth, invasion, or metastasis? The authors have generated an inducible model that should facilitate these studies. 4) Is the tumor-promoting activity of LMW-E due to enhanced deregulated kinase activity or to alternative target specificity? The LMW-E construct used in these studies has an N-terminal deletion (40 amino acids) that eliminates the proposed nuclear localization signal (NLS) and potentially affects the intracellular localization and substrate specificity [22], [23]. 5) How is LMW-E generated in tumors, and is it tumor-type specific? It has been proposed and demonstrated by Caruso et al. that many tumors have elevated protease activity and decreased levels of protease inhibitors such as elafin [24] that may contribute to the generation of LMW-E. Further characterization of the proteolytic pathways that target cyclin E in tumors may provide alternative therapeutic targets.
